# Hepatitis E virus infection in hemodialysis patients: A seroepidemiological survey in Iran

**DOI:** 10.1186/1471-2334-5-36

**Published:** 2005-05-17

**Authors:** Mahnaz Taremi, Manouchehr Khoshbaten, Latif Gachkar, MohammadJavad EhsaniArdakani, MohammadReza Zali

**Affiliations:** 1 National Research Department of Foodborne Diseases (NRDFD), The Research Center for Gastroenterology and Liver Diseases, Shaheed Beheshti University of Medical Sciences, Taleghani Hospital, Tabnak St., Evin, Tehran, Iran; 2 Drug Applied Research Center (DARC), Tabriz University of Medical Sciences, Tabriz, Iran; 3 Infectious Diseases and Tropical Medicine Research Center (IDTMRC), Shaheed Beheshti University of Medical Sciences, Tehran, Iran; 4 Gastroenterology and Liver Diseases Department, Shaheed Beheshti University of Medical Sciences, Tehran, Iran; 5 Research Center for Gastroenterology and Liver Diseases (RCGLD), Shaheed Beheshti University of Medical Sciences, Taleghani Hospital, Tabnak St., Evin, Tehran, Iran

## Abstract

**Background:**

The hepatitis E virus (HEV) has a global distribution and is known to have caused large waterborne epidemics of icteric hepatitis. Transmission is generally via the fecal-oral route. Some reports have suggested parenteral transmission of HEV. Anti-HEV prevalence data among chronic hemodialysis (HD) patients are few and give conflicting results.

**Methods:**

This cross-sectional study was conducted in August of 2004. We tested 324 chronic HD patients attending three different units in the city of Tabriz, northwestern part of Iran, for anti-HEV antibody. A specific solid- phase enzyme-linked immunoassay (Diapro, Italy) was used.

**Results:**

The overall seroprevalence of hepatitis E was 7.4 %(95% CI: 4.6%–10.6%). The prevalence rate of HBV and HCV infection were 4.6% (95% CI: 2.3%–6.9%) and 20.4% (95% CI: 16%–24.8%), respectively. No significant association was found between anti-HEV positivity and age, sex, duration of hemodialysis, positivity for hepatitis B or C virus infection markers and history of transfusion.

**Conclusion:**

We observed high anti-HEV antibody prevalence; there was no association between HEV and blood borne infections (HBV, HCV, and HIV) in our HD patients. This is the first report concerning seroepidemiology of HEV infection in a large group of chronic HD individuals in Iran.

## Background

The causative agent of hepatitis E, hepatitis E virus (HEV) had provisionally been classified into the caliciviridae family, but in the most recent ICTV (International Committee on Taxonomy of Viruses) classification, HEV has been placed in its own taxonomic group within the class IV (+) sense RNA viruses, "Hepatitis E-like viruses" [1,2].

Hepatitis E is an important public health concern in many developing countries of Southeast and Central Asia, the Middle East, northern and western parts of Africa, and Mexico, where the occurrence of outbreaks have been reported [3-7]. Although the overall mortality rate associated with HEV infection is low, it is reportedly as high as 20% in infected pregnant women [8,9].

Transmission of HEV occurs primarily by the fecal-oral route through contaminated water supplies in developing countries. Recent studies have indicated that zoonosis is involved in the transmission of HEV, especially in industrialized countries where hepatitis E had been believed to be non-endemic [10,11]. Further, vertical transmission of HEV from infected mothers to their children has been observed [12]. Also dental treatments were suspected as risk factors for HEV contamination [13]. It has been reported that a substantial proportion of blood donors (3/200 or 1.5%) were positive for HEV RNA and viremic blood donors are potentially able to cause transfusion-associated hepatitis E in areas of high endemicity [14,15]. Patients on chronic hemodialysis have an increased risk of exposure to nosocomially-transmitted agents, and the possibility of transmission of HEV in this group of patients had been raised. Reports on the prevalence and possible nosocomial transmission of HEV in patients on hemodialysis (HD) are scant. Some authors observed a high prevalence of anti-HEV antibody in their hemodialysis patients and hypothesized that the fecal-oral route may not be the only route of transmission of HEV among their patients [16]. Other investigators, in contrast, found few anti-HEV-positive patients in their HD populations [17,18]. Moreover, in most of these reports a small number of patients were studied.

To our knowledge, the seroprevalence rate of anti- HEV among HD patients in Iran has not been examined. Iran is a country with few suspected outbreaks of HEV [19]. These findings prompted the present study to establish the prevalence of anti-HEV antibody among HD patients in the city of Tabriz in the northwestern part of Iran.

## Methods

### Patients

This study was carried out in Tabriz, northwestern part of Iran with about 3.5 million residents. We studied all patients (n = 324) on chronic hemodialysis treatment at three different dialysis units in Tabriz in August 2004. Routine HD techniques were performed with 3 or 4-hours treatments three times a week. Blood samples were obtained from all the patients for serological testing. Information was obtained from the medical records of the patients on the duration of hemodialysis and etiology of renal failure. In addition, a questionnaire was completed for each subject detailing the age, sex, and history of blood transfusion.

### Laboratory assay

Blood samples were taken from each patient before the hemodialysis session, and the serum was separated without delay. Sera were stored at -20°C, coded and further tested at the laboratory of Research Center for Gastroenterology and Liver Diseases (Taleghani Hospital, Tehran) for anti-HEV IgG by enzyme immunometric assay (Diapro, Italy HEV EIA) according to the manufacturer's instruction. Cut-off was defined with positive and negative control sera that were included in each assay, according to manufacturer's instruction. Samples were considered positive if the optical density (OD) value was above the cut-off value and all positive samples were retested in duplicate with the same EIA assay to confirm the initial results. All patients were previously tested for HBs Ag (Diasorin, USA), anti-HCV (Third generation assay, Diasorin, USA) and anti-HIV (Biotest, Germany) by EIA assay.

### Statistical analysis

Statistical analysis was performed with SPSS (version 11) statistical software (Scientific Package for Social Sciences, Chicago, IL). Descriptive statistics were reported. Continuous variables were summarized as mean ± SD, and comparisons were performed with use of t-test for unpaired samples. The chi-square test, Fisher's exact test and Mann-Whitney test were used to compare the proportions between groups. The level of significance was set at a P value of <0.05. The study was endorsed by the responsible ethics committee.

## Results

Three hundred and twenty four patients were tested. There were 190 (59%) males, and 134 (41%) females; the mean age (± SD) was 53.5 ± 15.1 years. The median duration of HD treatment was 27 months (range1–261 months). The chronic renal failure of the patients was due to glomerulonephritis (n = 113), diabetic nephropathy (n = 73), nephroangiosclerosis (n = 28), polycystic kidney disease (n = 22), chronic interstitial nephritis (n = 5) and other etiologies (n = 83). No patient admitted a history of intravenous drug use.

There were twenty-four of 324 chronic hemodialysis patients showing anti-HEV antibody, the anti-HEV prevalence in our population was 7.4% (95%CI: 4.6%–10.6%). There were 15/324(4.6%) patients with persistent HBV infection (HBs Ag positive) and 66/324 (20.4%) patients were anti-HCV antibody positive. All the patients were seronegative for anti-HIV antibody. Table 1 shows the patient characteristics in relation to the presence of HEV coinfection. No statistically significant difference was seen between anti-HEV positive and negative patients with respect to age, sex, incidence of blood transfusion and other blood borne infections (HBV, HCV, and HIV).

**Table 1:** Characteristics of anti-HEV positive and negative patients in relation to HEV coinfection.

Characteristics	Anti-HEV positive (n = 24)	Anti-HEV negative (n = 300)	P
Age (year)^a^	58.7 ± 12	53 ± 15.3	NS
Sex (M/F)	15/9	175/125	NS
Incidence of blood transfusion	3.5 ± 6.6	2.5 ± 7	NS
HBV-positive patients	0/24 (0%)	15/300 (5%)	NS
Anti-HCV-positive patients	2/24 (8.3%)	64/300 (21.3%)	NS

HBV, hepatitis B virus; HCV, hepatitis C virus; HD, hemodialysis; NS, not significant.

^a^Expressed as the mean ± SD.

The duration of hemodialysis ranged between1 and 121 months (median 26) for anti-HEV positive patients and between 1 and 261 months (median 27) for the anti-HEV negative one with no significant difference (Mann-Whitney P = 0.73).

## Discussion

Studies concerning HEV epidemiology among chronic HD patients are few and give conflicting results. Difference of HEV prevalence in the general population, the criteria for inclusion of patients, and the routes of HEV transmission could partially explain the diverse results found.

HEV is usually associated with the fecal-oral route, and blood is not considered an important cause of HEV transmission as the virus dose not produces a chronic carrier state [3]. However, experimental transmission of HEV to man showed a transient phase of viremia proceeding the onset of clinical symptoms and prolonged viremia has been observed in some patients [20-22]. Therefore, a theoretical possibility of HEV transmission in endemic areas via a parenteral route has been suggested. Such a possibility is supported by the observation that anti-HEV antibody is more frequent in transfusion recipients than in the same number of non-transfused controls [23].

We found that the prevalence of anti-HEV IgG was 7.4 % in chronic HD patients attending three different units in the city of Tabriz, northwestern part of Iran. There were few reports on suspected outbreaks of HEV in Iran [19] and a population-based study indicated that the prevalence rate of IgG anti-HEV among healthy population was 9.6% [24]. The observation that anti-HEV prevalence rate in HD patients in our study (7.4%) is lower than in the previous general population-based study (9.6%)might be due to differences in the population, the size of the sample studied or situation of public health services. In Iran, large cities have better public health services, such as clinics, municipal water and sewage systems, possibly explaining the reduced risk of infection. These factors need to be further evaluated.

The detection of IgG anti-HEV in 7.4% of chronic HD provides additional evidence that HEV is endemic in Iran. Similar findings have been reported from Saudi Arabia [25], where 7.2% of HD patients were detected for IgG anti- HEV. In contrast, the prevalence of IgG anti-HEV in patients at the hemodialysis unit of non- endemic countries was significantly low [26-28]. The prevalence of HEV vary in different hemodialysis units, relatively independent of the prevalence in the general population and probably reflect the difference in the epidemiological characteristics of HEV in areas of low and high endemicity [27,29]. Apart from socioeconomic and environmental factors, intra-unit factors that may be associated with HEV transmission in some hemodialysis units needs to be evaluated further [29].

No statistically significant association was observed between HEV seropositivity and blood-borne viruses (HBV, HCV and HIV). One study has shown a striking association between hepatitis C and HEV, pointing to similar or overlapping routes of transmission [30]. Such an observation is in contrast with our finding. HEV infection, as detected by anti-HEV antibody, was associated with no risk factor in most patients.

## Conclusion

This cross-sectional study showed a high prevalence of anti-HEV antibody in our HD Patients; we didn't find association between HEV and blood-borne viruses.

A careful surveillance in the general population is required and further appropriate investigations are needed to identify the exact mode of transmission and risk groups for this infection in Iran.

## Competing interests

The author(s) declare that they have no competing interests.

## Authors' contributions

MT raised the original idea and design of the study and prepared the first draft of the manuscript. MK conceived the study, and participated in its design and coordination.

LG participated in the design of the study and performed the statistical analysis. MJE and MRZ revised the draft manuscript. All authors have read and approved the final manuscript.

## Pre-publication history

The pre-publication history for this paper can be accessed here:


